# Transcriptome analysis of embryo maturation in maize

**DOI:** 10.1186/1471-2229-13-19

**Published:** 2013-02-04

**Authors:** Keat Thomas Teoh, Deborah Vicuna Requesens, Shivakumar P Devaiah, Daniel Johnson, Xiuzhen Huang, John A Howard, Elizabeth E Hood

**Affiliations:** 1 Arkansas State University Biosciences Institute, PO Box 639, 72467, State University, AR, USA; 2 Applied Biotechnology Institute, San Luis Obispo, CA, USA

**Keywords:** Transcriptome, Maize, Embryo, Maturation, qRT-PCR

## Abstract

**Background:**

Maize is one of the most important crops in the world. With the exponentially increasing population and the need for ever increased food and feed production, an increased yield of maize grain (as well as rice, wheat and other grains) will be critical. Maize grain development is understood from the perspective of morphology, hormone responses, and storage reserve accumulation. This includes various studies on gene expression during embryo development and maturation but a global study of gene expression of the embryo has not been possible until recently. Transcriptome analysis is a powerful new tool that can be used to understand the genetic basis of embryo maturation.

**Results:**

We undertook a transcriptomic analysis of normal maturing embryos at 15, 21 and 27 days after pollination (DAP), of one elite maize germplasm line that was utilized in crosses to transgenic plants. More than 19,000 genes were analyzed by this method and the challenge was to select subsets of genes that are vitally important to embryo development and maturation for the initial analysis. We describe the changes in expression for genes relating to primary metabolic pathways, DNA synthesis, late embryogenesis proteins and embryo storage proteins, shown through transcriptome analysis and confirmed levels of transcription for some genes in the transcriptome using qRT-PCR.

**Conclusions:**

Numerous genes involved in embryo maturation have been identified, many of which show changes in expression level during the progression from 15 to 27 DAP. An expected array of genes involved in primary metabolism was identified. Moreover, more than 30% of transcripts represented un-annotated genes, leaving many functions to be discovered. Of particular interest are the storage protein genes, globulin-1, globulin-2 and an unidentified cupin family gene. When expressing foreign proteins in maize, the globulin-1 promoter is most often used, but this cupin family gene has much higher expression and may be a better candidate for foreign gene expression in maize embryos. Results such as these allow identification of candidate genes and promoters that may not otherwise be available for use. mRNA seq data archived in NCBI SRA; Accession number: ACC=SRA060791 subid=108584.

## Background

Our laboratory is using the maize embryo to express foreign proteins for industrial applications [1-3]. For example, genes for an endocellulase, E1, and an exocellulase, CBH I, have been transformed into maize, plants recovered, and seed collected. The original maize tissue culture germplasm, Hi-II, is transformation competent but not agronomically productive [4]. Thus, high-expressing transformants must be bred into elite germplasm for improved field performance to optimize the production system for commercialization. In each case, when original transformants are bred into elite germplasm, higher accumulation of the target protein can be achieved by selection [3,5,6]. While this has been observed empirically many times in corn, the mechanism of this phenomenon is not known. In order to understand the genetic basis of this mechanism, an understanding of the genes involved in normal embryo development is critical. The embryo maturation stage is critical for our studies because the foreign genes of interest, i.e. the cellulases, are expressed from the globulin-1 promoter—an embryo seed storage protein promoter that is active during the mid-maturation phase of embryo development [7].

The process of seed development in maize is understood from the perspective of morphology, storage protein accumulation and hormone responses [8-12]. For example, Kiesselbach [10] published one of the earliest treatises on the development of maize, including the gametes, embryo and seed. His was primarily a visual study using the light microscope with limited sub-cellular detail though elegantly detailed on the developmental timeline. In addition, Kriz [11] added detail on reserves by showing that the globulins (1 and 2) are the most abundant storage proteins in the embryo. These proteins are formed during maturation and degraded during germination, providing carbon and nitrogen sources for the growing seedling. In the endosperm, zeins are the major storage reserves and their accumulation is intricately staged [9]. These reserves are also degraded during germination to feed the growing embryo. McCarty [8] reviewed the viviparous mutants of maize and their phenotypic responses to abscisic acid (ABA) and gibberellins (GAs) during embryo development and maturation. ABA is best known for its control of plant responses to stress, including drought [13]. Similarly, dessication in seeds is controlled by ABA [8]. Collectively these studies describe a framework that defines seed development and maturation. However, to fully understand these processes, we must take advantage of the new technologies that are available, such as transcriptome sequencing.

Specifically for the embryo, Kiesselbach [10] showed that at 13–15 days after pollination (DAP) the embryo has only the rudimentary structures of a scutellum (cotyledon) and meristem. By 21 DAP, the embryo structure is apparent with the shoot and root tips discernible. By 25 DAP, the embryonic structures are fully formed with shoot tip and radical covered by the coleoptile and the coleorhiza, respectively [10]. According to Vernoud et al. [8], the embryo starts maturation at 15 DAP and storage reserves are accumulating by 21 DAP. We used these early studies to design transcriptome sequencing experiments of maturing embryos.

Previous gene expression studies describe the regulation of seed development and maturation, including patterns for cell division, DNA replication, induction of morphological changes, storage protein accumulation and desiccation for dormancy [14-17]. Vernoud et al. [15] provide an overview of gene expression during maize embryogenesis, including descriptions of mutants and cloned genes. Microarray profiling of gene expression changes during embryo development was performed by Lee et al. [14]. Their custom array included 900 genes from EST libraries that were predicted to be involved in metabolism and embryogenesis. They found groups of genes that were expressed at different stages during seed development, moving from cell division activities at early stages to storage reserve synthesis and dessication. Genome-wide microRNA genes are also being surveyed to eventually determine their functional role in regulation of seed growth and development [17].

We undertook a transcriptomic analysis of normal maturing embryos of one elite germplasm line that is utilized for the crosses to transgenic events originally made in Hi II germplasm. In this study, we analyzed the transcriptome of embryos from 15, 21 and 27 DAP to determine the genes that are expressed at these time points. More than 19,000 genes were analyzed by this method and the challenge was to choose subsets of genes that are vitally important to embryo development and maturation for the initial analysis. We describe the changes in expression of genes relating to primary metabolic pathways, DNA synthesis, late embryogenesis proteins, and embryo storage proteins. We describe changes shown through transcriptome analysis and confirmed expression levels of a subset of genes in the transcriptome through qRT-PCR.

## Results

### Collection of embryos

**Figure 1:**
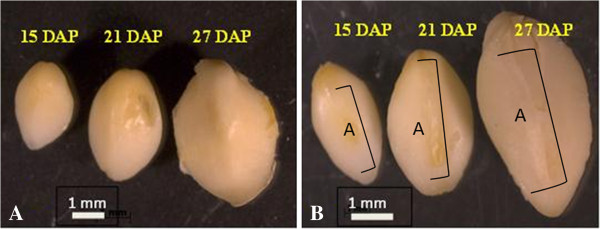
**Maize embryos removed from seeds at 15, 21, and 27 days after pollination (DAP). A**. Scutellar surface; **B**. Embryonic axis. Limits of axis [A] are indicated by brackets. Bar = 1 mm.

The elite inbred, SP114, from which the embryos were analyzed is a Stiff Stalk variety germplasm (USP #6,252,148). A parallel Lancaster variety is used as a complement for the hybrid but is not described here. Embryos were isolated under sterile conditions using immature ears from plants grown in the greenhouse with 16 hour light and 8 hour dark periods. We have chosen to study the expression of maize embryo genes at three time points, 15, 21 and 27 days after pollination (DAP), referred to as S15, S21 and S27, combining the genotype (SP114) with the harvest time. We were interested in gene expression changes over the greatest range of active maturation, and these three times correspond to the early, mid and late phases of embryo maturation (Figure 1). The first observation is the dramatic change in size during this time period. Clearly, much growth has occurred between 15 and 27 DAP, as well as significant development of the embryonic axis. We wanted to analyze embryos at stages encompassing storage protein accumulation because the promoter that drives the expression of our transgenes is the globulin-1 promoter, an embryo storage protein. The three time points chosen correspond with the expression pattern of the globulin-1 gene. The transcripts of the globulin-1 gene have been shown to begin accumulating at approximately 18 DAP, peak at 24 DAP and begin to decline at 27 DAP [7].

### Overview of the maize embryo transcriptome

Total RNA was isolated from pooled maturing embryos from a single ear at each sampling time. RNA sequencing was performed using Illumina GA II/Solexa instrumentation (Tufts Core Facility, Tufts University School of Medicine, Boston, MA). Single end reads of seventy-two nucleotides were conducted. A total of 56.2 million raw reads were generated for the three samples, out of which 52.9 million trimmed reads were available for mapping to the maize reference genome ZmB73AGPv1 (Table 1). The number of trimmed reads actually mapped to the reference genome was 42.9 million but only 10.4 million of these reads (20%) were uniquely mapped to individual loci. Almost 70% of the uniquely mapped reads were mapped within known exons. The summary of the trimming and alignment for each sample is shown in Table 1.

**Table 1:** Summary of trimming and alignment

**Samples**	**Raw reads**	**Trimmed reads**	**Mapped reads (%, mapped/trimmed)**	**Uniquely mapped reads (%, unique/trimmed)**	**Uniquely mapped reads in genes (%, in genes/unique)**
S15	22,449,515	20,907,381	16,987,823	4,078,452	2,879,805
			(81.3%)	(19.5%)	(70.6%)
S21	19,673,902	18,922,999	14,712,171	3,950,666	2,801,575
			(77.7%)	(20.9%)	(70.9%)
S27	14,104,109	13,104,891	11,217,261	2,405,976	1,577,049
			(85.6%)	(18.4%)	(65.5%)
Total	56,227,526	52,935,271	42,917,255	10,435,094	7,258,429
			(81.1%)	(19.7%)	(69.6%)

**Figure 2:**
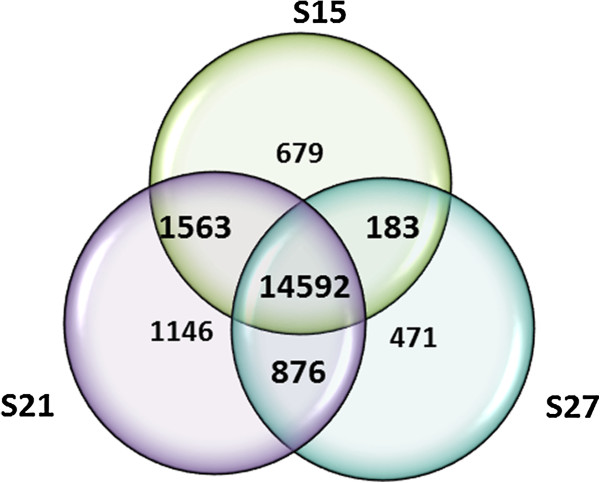
**Shared and unique genes (RPKM ≥ 1) among the three maturing stages of the maize embryo.** 19,510 genes are expressed at the 3 stages with the majority (14,592) expressed at all stages. S15 = 679 unique genes; S21 = 1146 unique genes; S27 = 471 unique genes.

**Figure 3:**
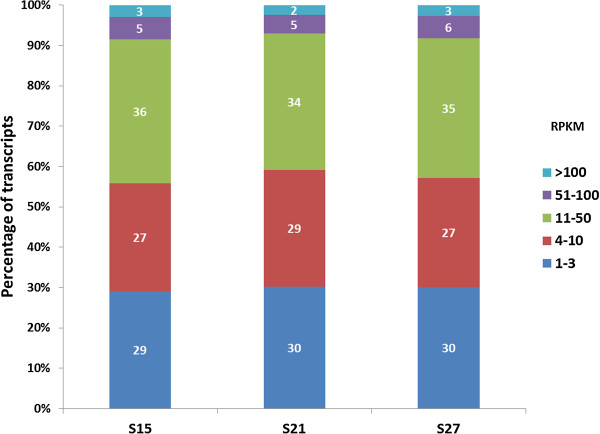
**Percent of transcripts with different expression abundances based on RPKM.** Genes showing 11–50 copies per transcriptome were most highly represented at each stage.

The normalized reads referred to as reads per kilobase of exon per million mapped reads (RPKM), were used to estimate the total number of genes expressed throughout embryo maturation. The RPKM method corrects for biases in total gene exon size and normalizes for the total number of read sequences of each library obtained from each sample [18]. RPKM values ≥ 1 were used in the estimation of the number of genes expressed. The total number of genes counted in the maturing embryo was 19,510 representing almost 60% of the annotated transcriptome of maize. Of these, 17,017 (87%) were expressed in S15, 18,177 (93%) in S21 and 16,122 (83%) in S27 (Table 2). Figure 2 shows the number of genes uniquely expressed in each stage, or genes that are shared with one or two other stages. In this study, 14,592 (75%) of the expressed genes are represented in all three stages of embryo maturation that we analyzed and 12% are expressed in a single stage. The differences in expression of shared genes are of interest to discover how they change throughout embryo maturation. Moreover, single genes are of interest because of their potential importance at that stage. The gene expression levels in each stage of embryo maturation are classified into five categories based on their RPKM values – very low (1–5 RPKM), low (>5-10 RPKM), moderate (>10-50 RPKM), high (>50-100) and very high (>100 RPKM) (Figure 3). Only a small percentage of the genes fall into the high (5-6%) and very high abundance (2-3%) categories.

**Table 2:** Number of genes expressed at each stage of embryo maturation

**Stage**	**Total number of genes expressed**
S15	17,107
S21	18,177
S27	16,122
Average	17,105

### Differential expression of maize embryo genes

**Figure 4:**
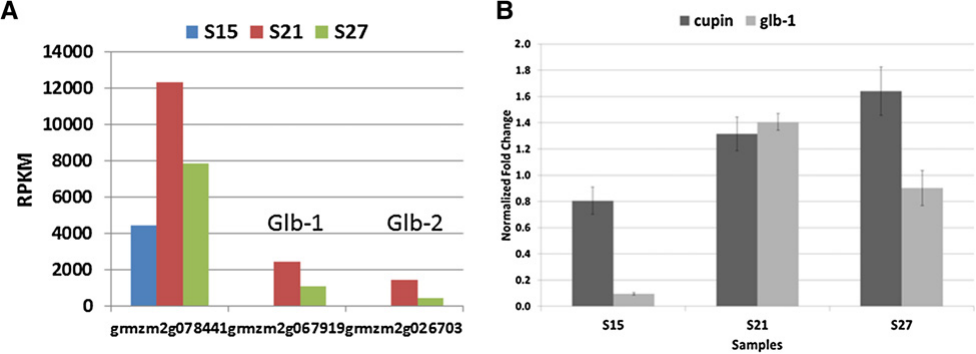
**Transcript profile (A) and qPCR results (B) of cupin family storage protein genes in maturing maize embryos. A**. Each set of 3 vertical bars (blue, red, green) indicates the number of transcripts counted for the respective time points for the individual locus enumerated on the X-axis. **B**. qPCR counts for glb-1 and cupin genes.

The number of genes that showed differential expression (p < 0.001 and q < 0.001) is 7,124 out of the 19,510 total genes expressed in maturing embryos, representing approximately 36.5% of the embryo transcriptome. More than half of the differentially expressed genes showed at least a two-fold change in expression level in all three pairwise comparisons (Table 3). For genes that showed at least a two-fold change, almost as many were up-regulated as down-regulated, except between S27 and S15, where a higher proportion of genes were down-regulated, 56% compared to 44% that were up-regulated (Table 3). The proportion of differentially expressed genes was highest in the S27 vs S15 comparison (46%) and lowest in the S21 vs S27 comparison (36%) (Additional file 1: Figure S1). We used qRT-PCR to validate the expression levels of 11 transcripts and found a high correlation (R^2^ = 0.941) between mRNA-seq data and qRT-PCR (Additional file 2: Table S1). The most notable result from the qRT-PCR was the high level of cupin expression compared to the globulin-1 gene during storage protein accumulation. (See Figure 4 for details).

**Table 3:** Differential expression of genes between pairwise comparisons of embryo stages

**Comparison stages**	**No. of genes tested**^**1**^	**Differentially expressed**^**2**^	**│log**_**2**_**FC│**^**3**^**≥ 1**	**Up-regulated (≥ 1)**	**Down-regulated (≤ 1)**
S15 vs S21	12,186	4,890	2,812	1,481	1,331
S21 vs S27	11,157	4,032	2,499	1,243	1,256
S27 vs S15	11,049	5,085	3,590	1,585	2,005

[1] Number of genes with 50 total reads combined from the two data sets in the pairwise comparison.

[2] p-value < 0.001 and q-value < 0.001. [3] Absolute value of log_2_ (fold change).

Embryo maturation follows embryo pattern formation and differentiation, beginning at 15 DAP and lasting for about 30 days [15]. Embryo maturation is marked by growth, active accumulation of reserve substances and some developmental events. Growth is characterized by a period of active precursor biosynthesis, DNA synthesis and cell division [19,20], and reserve accumulation is characterized by the biosynthesis and deposition of storage proteins, fatty acids and starch [21-23].

**Figure 5:**
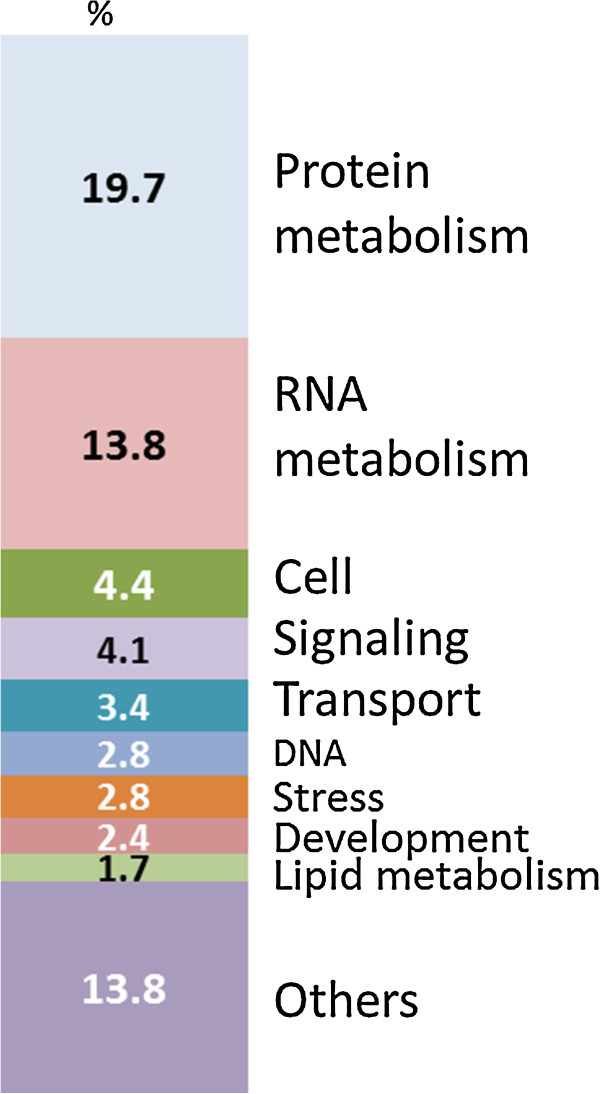
**Functional distribution of genes in maturing embryo.** Categories are based on those present in Mapman software. Approximately 35% of all genes expressed are involved in protein and RNA metabolism.

We adopted Mapman annotation for the SP114 embryo transcriptome using the mapping file Zm_Genome_Release_09 [24,25] to assign genes to 35 functional categories (Additional file 3: Table S2). Protein and RNA metabolism are the two functional categories showing the greatest number of transcribed genes. The distribution of normalized reads among the top nine functional categories is shown in Figure 5 excluding the 30% belonging to the ‘not assigned or unknown’ category (category 35).

**Figure 6:**
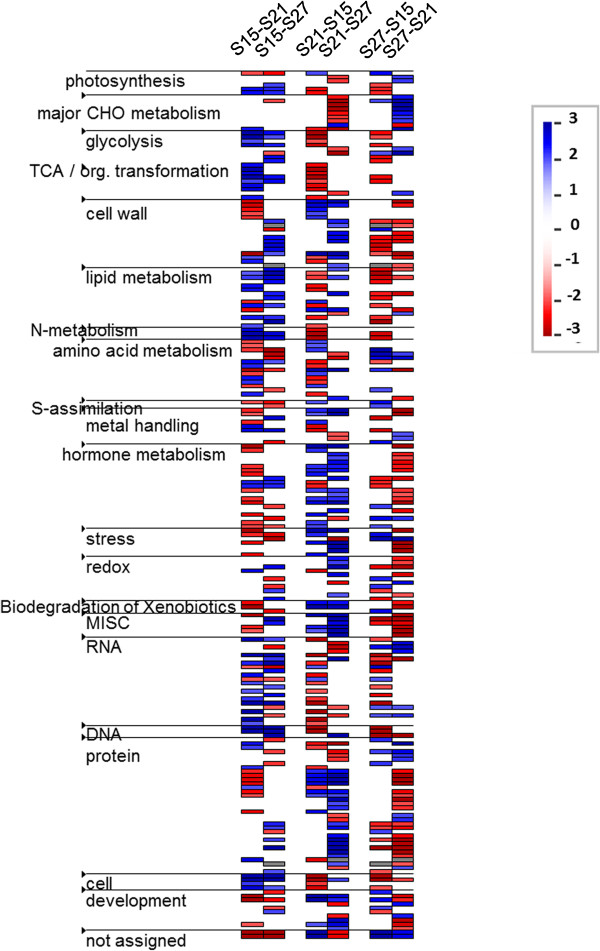
**Enriched functional categories in each of the pair-wise comparison.** Each vertical column represents the genes that are dramatically up (blue) or down (red) regulated when comparing the 2 sampling times indicated at the top. Lines to the left show each functional category derived from the Mapman software. Each color bar represents an individual locus.

We used PageMan [26] to obtain a statistics-based overview of enriched functional categories in each of the three pairwise comparisons (S15 vs. S21, S21 vs S27 and S27 vs S15). The transcriptome data were loaded into PageMan and a Wilcoxon test [27] was applied to each category. The Wilcoxon test compares the log base 2 fold change values of genes in a functional category against all genes not in that category. This reveals whether the genes in a particular category behave differently compared to all the other genes. This analysis condensed and compressed the genes by removing categories that did not show a significantly different change and displaying the categories that did show significant change using a false color heat-map-like display to show up- or down-regulated classes. Visual display of the Wilcoxon test results revealed enriched specific functional categories in each of the three pairwise comparisons (Figure 6). Genes encoding enzymes for light independent photosynthetic reactions, glycolysis, TCA cycle, lipid metabolism, RNA and DNA synthesis and cellular functions are strongly up-regulated during the early stage of embryo maturation (S15). At 21 DAP, gene enrichment is shifted to functional categories that include cell wall, metal handling, hormone metabolism, stress, biodegradation of xenobiotics and synthesis of storage proteins. At the later stage of embryo maturation (S27), genes in almost all the functional categories are down-regulated with the exception of major carbohydrate metabolism, sulfur assimilation and sub-classes of amino acid metabolism, RNA and protein.

#### Glycolysis

**Figure 7:**
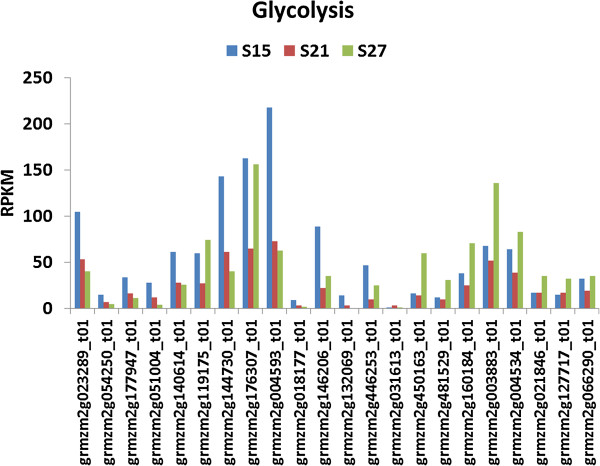
**Transcript profile of genes assigned to Glycolysis in maturing maize embryos.** Each set of 3 vertical bars (blue, red, green) indicates the number of transcripts counted for the respective time points for the individual locus enumerated on the X-axis.

**Figure 8:**
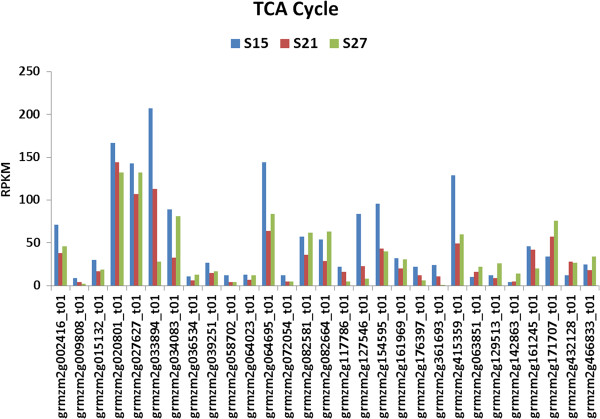
**Transcript profile of genes assigned to the TCA Cycle in maturing maize embryos.** Each set of 3 vertical bars (blue, red, green) indicates the number of transcripts counted for the respective time points for the individual locus enumerated on the X-axis.

Glycolysis and the mitochondrial TCA cycle are important pathways in embryo maturation. They provide the energy required for active growth. Transcripts assigned to these two pathways showed two distinct groups of genes (Figures 7 &8). The first group had high expression during the early stage of embryo maturation and was characterized by high overall levels of transcript accumulation. The second group was up-regulated at 27 DAP and is characterized by much lower relative levels of transcript accumulation. Almost all the genes in glycolysis and the TCA cycle appeared to have minimal expression levels at 21 DAP (Figure 7 and Figure 8).

#### Lipid metabolism

**Figure 9:**
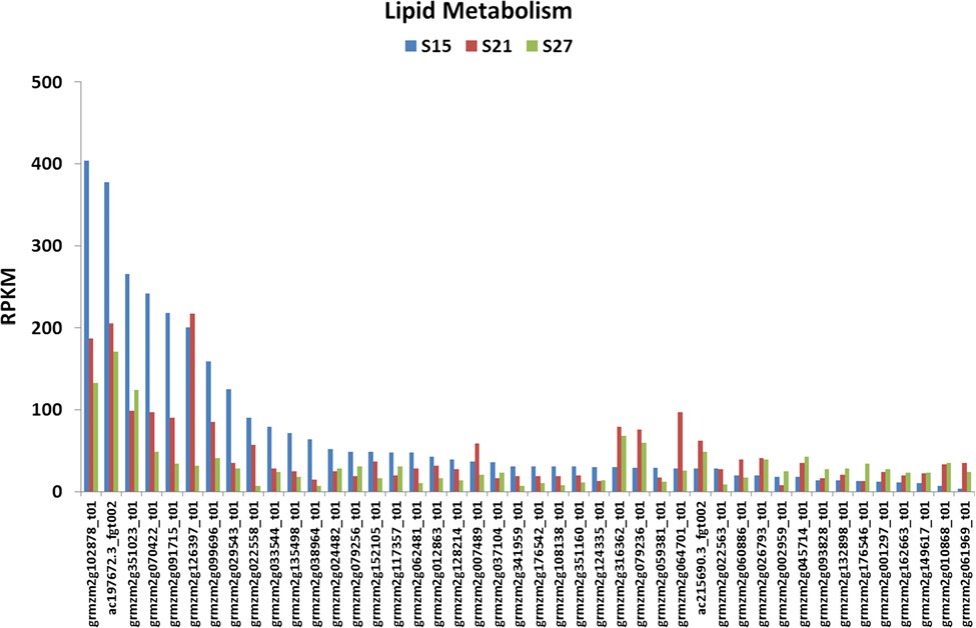
**Transcript profile of genes assigned to Lipid Metabolism in maturing maize embryos.** Each set of 3 vertical bars (blue, red, green) indicates the number of transcripts counted for the respective time points for the individual locus enumerated on the X-axis.

The embryo is the site of active fatty acid production in the kernel. Lipids can accumulate at up to 50% of the dry weight of the maize embryo at maturation [28]. Active fatty acid biosynthesis occurs early in embryo maturation and continues to about 21 DAP. Three groups of genes are assigned to lipid metabolism (Figure 9). The first comprises about 25 genes that are induced early during maturation. They are associated mainly with fatty acid synthesis and fatty acid elongation. This group is characterized by a higher overall transcript accumulation than the other two groups. A second smaller group of genes induced at 21 DAP comprises genes that encode a variety of enzymes associated with lipid modification such as ACP desaturases and biosynthesis of triacylglycerol (TAG). The third group of genes comprises a few that are associated with biosynthesis of sphingolipids, lipids derived from isoprenoids such as sterols and squalene, as well as with lipid degradation.

**Figure 10:**
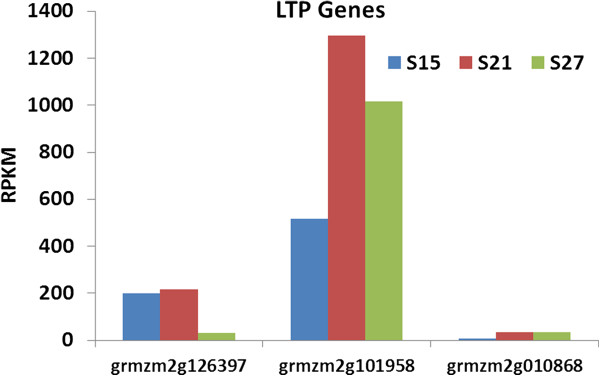
**Transcript profile of Lipid Transfer Protein genes in maturing maize embryos.** Each set of 3 vertical bars (blue, red, green) indicates the number of transcripts counted for the respective time points for the individual locus enumerated on the X-axis.

Three genes are assigned to lipid transfer proteins (LTPs). Lipid transfer proteins have been shown to facilitate *in vitro* transfer of lipids between membranes and are assumed to play a role in membrane biogenesis [29]. Two of the genes, LTP1 and LTP2, are expressed at very high levels but their expression patterns are different from each other. The accumulation of the LTP1 transcript, grmzm2g126397_t01, was high at 15 DAP and continued through 21 DAP but dropped sharply by 27 DAP (Figure 10). On the other hand, the accumulation of the LTP2 transcript*,* grmzm2g101958_t01, started at a high level at 15 DAP, increased dramatically at 21 DAP and maintained through 27 DAP. LTP3 is expressed at a low to moderate level and exhibited a similar expression pattern to LTP2.

#### DNA synthesis

**Figure 11:**
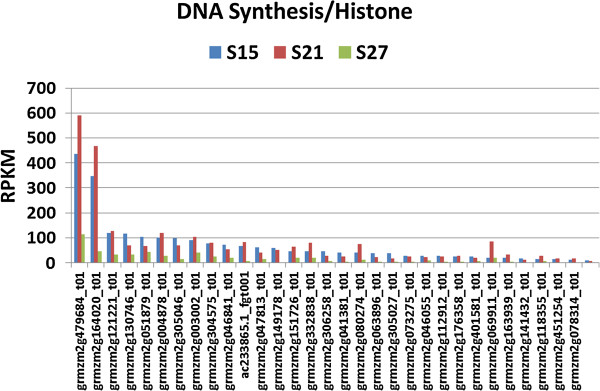
**Transcript profile of genes assigned to DNA Synthesis/Histone.** Each set of 3 vertical bars (blue, red, green) indicates the number of transcripts counted for the respective time points for the individual locus enumerated on the X-axis.

DNA synthesis related genes such as those controlling chromatin structure and histone modification, are high in the early stages of embryo maturation (Figure 11), reflected by the large number of genes and the relatively high level of their transcripts in the S15 and S21 embryos, either declining sharply by 27 DAP or in some cases no longer detected at all. A number of genes associated with chromatin structure showed high expression at 27 DAP (Additional file 4: Figure S2) but none are histone genes. Two histone genes, grmzm2g164020 and grmzm2g479684 that encode histone H1 and histone H4 respectively, are expressed at an exceptionally high level throughout the embryo maturation period in particular at 15 and 21 DAP (Figure 11).

#### Development

**Figure 12:**
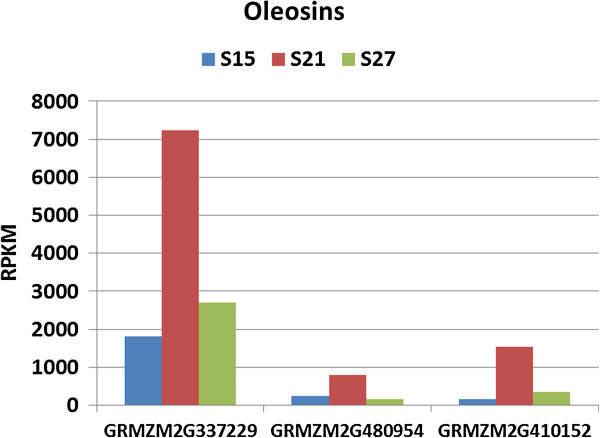
**Transcript profile of Oleosin genes in maturing maize embryos.** Each set of 3 vertical bars (blue, red, green) indicates the number of transcripts counted for the respective time points for the individual locus enumerated on the X-axis.

**Figure 13:**
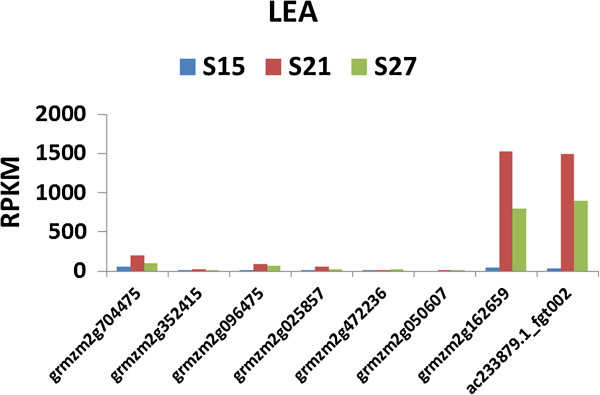
**Transcript profile of Late Embryogenesis Abundant genes in maturing maize embryos.** Each set of 3 vertical bars (blue, red, green) indicates the number of transcripts counted for the respective time points for the individual locus enumerated on the X-axis.

Genes assigned to the development category code for storage proteins of the cupin family which include the globulin proteins, oleosins, and late embryogenesis abundant (LEA) proteins (Figure 4, Figures 12–13). In contrast to genes involved in DNA synthesis and cellular function, genes assigned to the development category showed low expression at 15 DAP, peaked at 21 DAP and then declined slightly as the embryos entered the later maturation stage. Transcripts of genes within this category accumulate to an exceptionally high level at 21 DAP.

Three prominent genes that encode storage proteins in the embryo, grmzm2g067919 (*glb1*), grmzm2g026703 (*glb2*) and grmzm2g078441, a gene in the cupin family of unknown function, are highly expressed (Figure 4). The transcripts of these three genes taken together constitute about 99% of the transcripts of all storage protein genes. The globulin proteins are recognized as the major storage proteins in the mature embryo [11]. However, the transcriptome data suggest that their expression levels are not as high as grmzm2g078441. The transcript level of grmzm2g078441 even at its lowest (15 DAP) is higher than the peak levels of *glb1* and *glb2* (Figure 4A). Because of our interest in embryo expression of foreign genes, we performed quantitative PCR to confirm the high levels of these critical genes from the transcriptome. As shown in Figure 4B, expression level of the unknown cupin gene is 8-fold higher than glb-1 at 15 DAP, approximately equal at 21 DAP and 65% higher at 27 DAP. Although the qPCR results do not exactly mirror the transcriptome results, this unknown cupin gene holds interest for further analysis.

The oleosins represent the lipid storage proteins that are part of the structural unit of the single layer membrane surrounding lipid bodies in seed. Seven genes are assigned to the oleosins and three are highly represented in the maturing embryo transcriptome (Figure 12). The most highly represented oleosin gene is grmzm2g337229 (oleosin 1), which at 21 DAP represents 75% of all the oleosin transcripts in the transcriptome.

Among the eight LEA genes in our transcriptome data, two stood out very prominently. These two LEA transcripts, grmzm2g162659_t01 and ac233879.1_fgt002, are homologs of Arabidopsis ATEM1 and ATEM6, respectively. These two LEA transcripts increased dramatically (30- to 50-fold) from 15 DAP to 21 DAP. The LEA transcripts have been shown to be inducible by abscisic acid (ABA) but do not require *vp1*[30,31]. The transcripts of the other LEA genes do not accumulate to a significant level (Figure 13).

### Gene ontology

**Figure 14:**
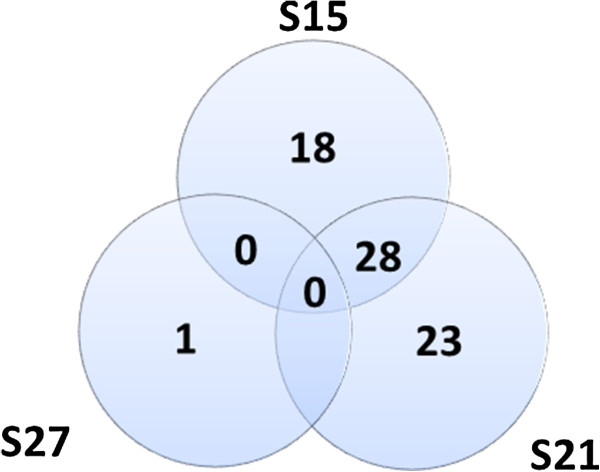
**Shared and Unique Gene Ontology Terms.** S15 = 18 unique gene ontology terms; S21 = 23 unique gene ontology terms; S27 = 1 unique gene ontology term. There is no gene ontology term that is shared among all the three stages, between S15 and S27 and between S21 and S27.

Analysis of the Gene Ontology (GO) terms represented in the maize embryo transcripts revealed significantly over-represented GO terms that are unique to each of the embryo maturation stages as well as GO terms that are shared between S15 and S21 (Figure 14). The most notably enriched GO terms that are unique to S15 are microtubule associated complex, microtubule motor activity, microtubule-based movement, motor activity, microtubule-based process, cytoskeletal part, fatty acid metabolic process, fatty acid biosynthetic process and lipid biosynthetic process. As for S21, the most notably unique GO terms are cellular components and cellular component organization, biological process and molecular functions associated with proteins such as protein oligomerization and protein-DNA complex assembly as well as GO terms associated with response to stress. There is only one GO term that is significantly enriched in S27 and that is nutrient reservoir activity. A list of the descriptions for the GO terms is presented in Additional file 5: Table S3.

### Single nucleotide polymorphism (SNP) discovery

Maize is generally considered highly polymorphic with a relatively high frequency of SNPs. The high frequency of SNPs coupled with improvements in sequencing technology and high throughput genotyping methods like DNA chips, allele-specific PCR and primer extension approaches, have made SNPs especially attractive as genetic markers [32] for discovering genes and identifying germplasm. Table 4 shows the numbers of SNPs discovered per chromosome. The discovered SNPs and their context sequences are provided in Table 4. The highest numbers of SNPs lay in chromosomes 1 and 3 with 4,076 and 3,382 SNPs, respectively, whereas the lowest number of SNPs lays in chromosome 8 (717).

**Table 4:** Numbers of SNPs discovered per chromosome and per genotype via comparison to the reference genome

**Chr**	**S21**	**(%total)**
0^1^	76	(0.4)
1	4,076	(20.3)
2	1,664	(8.3)
3	3,382	(16.9)
4	1,926	(9.6)
5	1,586	(7.9)
6	1,945	(9.7)
7	2,116	(10.6)
8	717	(3.6)
9	1,136	(5.7)
10	1,408	(7.0)
Total	20,032	

[1] SNPs in sequences that have not been mapped to a chromosome.

## Discussion

Transcriptomics is a powerful tool to analyze gene expression within any living system. The data generated in a single experiment will answer many questions about the system of interest. These data will also generate a host of new questions that can be explored in more detail using these same techniques on related samples. The drawback to this generation of megadata sets is that the analysis of the data requires large computing capability, multiple software packages and decisions based on value judgments about how to parse the data into package sizes that make sense and can be interpreted biologically.

The Mapman Pathway and Pageman programs have allowed us to observe changes in gene expression patterns that correlate with the maturing phases of the maize embryo. At 15 DAP, the embryo is at the transition between late development and early maturation, therefore many of the genes associated with the early events of embryo development are beginning to wane in their expression levels. For examples, genes encoding precursor biosynthesis (lipids, amino acids), DNA and RNA synthesis, as well as cell division and organization functions are more highly represented at 15 DAP than at later stages (Figure 5). At 21 and 27 DAP which are firmly within the embryo maturation phase, gene expression for activities such as growth and build-up of reserves rose, while genes from the earlier phase dropped. These results are similar to those observed by Lee et al. [14] using microarray techniques. The advantage of transcriptomics over microarrays is the breadth of the discovery potential with RNA sequencing, in contrast to microarrays which are based on a limited number of gene sequences.

Almost 30% of the genes in our transcriptome do not have functions assigned to them. Many of these genes are unique to the specific stage of the embryo while others showed big differences in the level of expression in a pairwise comparison. Some of these uncategorized genes such as grmzm2g409101 and grmzm2g075042 are expressed at very high levels and are coordinately expressed with globulin-1 genes (data not shown). Further functional analysis will provide deeper insight into the roles these genes play in the maturation of the embryo or in the accumulation of storage proteins, roles that will be identified through mutations and network prediction and manipulation. Our transcriptome data also showed that the total number of expressed genes was highest at 21 DAP and decreased as the embryos increased in maturity. This is in agreement with data published by Lee et al. [14] and Luo et al. [33] who showed accumulation of individual mRNAs during maize kernel development were much lower after 25 DAP. The 21 DAP embryo also represents a transition stage from 15 DAP to 27 DAP and a large number of genes were shared between 21 DAP and the other two stages. Davidson et al. [34] found 22,493 genes expressed in 25 DAP B73 embryos, very near the number that we discovered in maturing embryos from SP114.

We focused on several sets of genes to show the utility of our data in understanding the gene expression changes at specific time points of embryo maturation. For example, the 2 histone genes that encode histones H1 and H4 showed very high expression throughout maturation, suggesting a very important role for these two genes during this critical time. In contrast, the LEA genes have been proposed to play a role in desiccation tolerance [35,36] which probably accounts for their increase in expression level later in embryo maturation. We are also interested in the accumulation of storage proteins in the maize embryo because understanding the expression pattern of these genes may help us understand how these genes are regulated. One of our most interesting observations relates to genes in the cupin family that encode storage proteins within the embryo. In our bio-factory experiments, we use the globulin-1 promoter [7] to drive expression of foreign genes for industrial enzyme production [3,5]. The goal of the bio-factory production system is to increase foreign protein accumulation by as much as possible to lower the cost of production. Thus, the observation of a transcript in the cupin family, unknown gene grmzm2g078441 in Figure 4, that shows significantly higher expression than the globulin-1 gene (grmzm2g067919) at 15 and 27 DAP, suggests that its promoter would be a more effective promoter for foreign gene expression. Belanger and Kriz [7] found glb-1 and 2 to be the most abundant proteins in the embryo. The question is why the cupin transcript appears to be expressed at a higher level than the globulins if this is the case. One explanation could be that the transcript does not produce a protein, but is a type of pseudo-gene. Another explanation is that the studies were performed on different types of maize, W64A and Va26 in the 1991 study, and SP114 in this study. In addition, globulins are produced by a multigene family and possibly were not fully accounted for by the alignment, whereas the cupin gene transcript could be higher, though the protein not as abundant. Each of these possibilities could be addressed in further studies.

“The GO project has developed three structured controlled vocabularies (ontologies) that describe gene products in terms of their associated biological processes, cellular components and molecular functions in a species-independent manner” (http://www.geneontology.org/GO.doc.shtml). This useful tool allows cross-species comparisons of gene functions because of the uniformity of gene annotation language. It also reduces complexity of gene expression categories to 3 functional categories in order to find unique functions in a particular transcriptome. A GO term that stands out as highly over-represented is notable for its activity and describes the basic functions going on at that particular time and place. When GO analysis was applied to the embryo maturation transcriptome, a few terms were found that were unique or shared at each stage (Figure 14). These terms will drive some interesting analysis in the future.

Alexandrov *et al.*[16], through large scale sequencing of maize cDNAs, showed the distribution of mRNA characteristics associated with their promoters, transcriptional start site predictors, and GC content, especially in the third position of the codon. It would be interesting to understand if the genes expressed in a particular tissue at a particular time differ in their specific structural characteristics based on these authors’ analysis. For example, groups of genes in one of the GC content modal groups may be preferred in one or another tissue or developmental event.

The results reported here provide a baseline for further studies on individual genes or groups of genes that will elucidate how a corn embryo matures and begins to shut down for dormancy. Results from those types of studies can be utilized to enhance genes that would improve yield for increased food or feed productivity. In this world of ever-increasing populations, such outcomes will be critical.

## Conclusions

Numerous genes involved in embryo maturation have been identified, many of which show significant changes in expression level during the progression from 15 to 27 DAP. An expected array of genes involved in primary metabolism was identified. Of particular interest are the storage protein genes, globulin-1, globulin-2 and an unidentified cupin family gene. When expressing foreign proteins in maize, the globulin-1 promoter is most often used, but this cupin family gene has much higher expression and may be a better candidate for foreign gene expression in maize embryos. Results such as these allow identification of candidate genes and promoters that may not otherwise be available for use. The transcriptome data show patterns of expression of different genes involved in embryo development and storage protein accumulation. The transcriptome data will also serve as valuable resources for functional characterization of maize genes as more than 30% of transcripts represented un-annotated genes, leaving many functions to be discovered. As genes in Arabidopsis and other model systems are annotated, the identification of some of these novel genes will be accomplished.

## Methods

### Plant materials and growth conditions

The maize (*Zea mays* L) inbred SP114 (USP #6,252,148) was grown in the greenhouse at the Arkansas Biosciences Institute in Metro Mix 200 (SunGro Horticulture, Bellevue, WA) soilless medium and fertilized with Osmocote. Temperature and light cycles were set at 27°C to 31°C for 16-h light and 20°C to 24°C for 8-h dark. The embryos were isolated at 15, 21 and 27 days after pollination (DAP) under aseptic conditions, frozen in liquid nitrogen and stored at −80°C until used for RNA extraction.

### RNA extraction

The frozen embryos were ground into a fine powder in liquid nitrogen and homogenized in TRI Reagent solution (Ambion, Austin, TX). Total RNA at 15, 21 and 27 DAP was isolated following the RNA Isolation protocol from Invitrogen (Carlsbad, CA) and purified using Qiagen RNAeasy Mini Spin Columns (Qiagen, Valencia, CA). The concentration and purity of the total RNA were determined using an ND-1000 Spectrophotometer Nanodrop system (Thermo Scientific, Wilmington, DE) as well as RNA gel electrophoresis (Formaldehyde buffer system).

### cDNA library construction and transcriptome sequencing

cDNA library construction and sequencing of the transcriptome were contracted to Tufts Core Facility at Tufts University School of Medicine, Boston, MA. The cDNA libraries were constructed following the procedures outlined in the manufacturer’s manual (Illumina, Inc, San Diego, CA). The sequencing of the transcriptome was done using the Illumina Genome Analyzer II /Solexa (Illumina, Inc, San Diego, CA). The cDNA library for the 21 DAP (S21) samples was run in a single Illumina flow cell lane while the cDNA libraries for 15 DAP (S15) and 27 DAP (S27) were each paired with a transgenic sample of the same age. The number of trimmed reads was 11.6 million for S15, 18.8 million for S21, and 15.4 million for S27. Single-end reads were obtained with ranges in length from 64 (S15 and S27) to 66 (S21) bases.

### Alignment of reads to the genome and data analysis

#### Mapping of RNA-seq reads

Raw reads were trimmed to remove low-quality nucleotides via a custom Data2Bio (Ames, Iowa) trimming script. GSNAP (Genomic Short-read Nucleotide Alignment Program, version 2010-07-37) [37], which allows for gapped alignments, including intron-spanning alignments, was used to map trimmed reads to the reference genome. Only reads with one unique best match in the reference genome and ≤ 2 mismatches every 36 bp, and ≤ 3 bp tails were used for subsequent analyses. The read depth of each gene was computed based on the coordinates of mapped reads and annotated locations of genes in the reference genome.

All reads were aligned to the reference genome for *Zea mays*, ZmB73AGPv1; Mitochondrian (AY506529.1) and Chloroplast (X8563.2). The alignment and initial analysis of the transcriptome data were done by Data2Bio (Ames, Iowa). Further analysis was conducted in-house using CLC Genomics Workbench (Cambridge, MA). Visualization of the mapping and pathways was carried out using publicly available software including Mapman (http://mapman.gabipd.org/) [38] and Integrated Genomic Viewer (IGV) (http://www.broadinstitute.org/software/igv/) [39].

#### Identification of differentially expressed genes via Fisher’s exact test

Normalization was conducted using a method that corrects for biases introduced by RNA composition and differences in the total numbers of mapped reads in the two samples [40]. Normalized read counts were used to calculate fold-changes (FC) and statistical significance. Fisher’s exact test was used to test the null hypothesis that expression of a given gene is not different between the two samples. Only genes having at least 50 mapped reads from the two samples combined were tested. Genes identified as candidates for differential expression were further filtered by correcting for multiple testing [41] and a false discovery rate of 0.1% (q-value). Statistically significant variation can be a consequence of either biological or technical variation in gene expression between the two samples.

#### Gene ontology (GO) analysis

The software goatools (https://github.com/tanghaibao/goatools) was used to perform the GO analysis. Over- and under-representation of certain GO terms were determined based on Fisher’s exact test. Two multiple correction controls (Bonferroni and permutation to control false discovery rate) [42] were implemented.

#### Single nucleotide polymorphism (SNP) discovery

SNPs were called via comparisons to the reference genome, ZmB73AGPv1. Sequence variants identified by Genomic Short-read Nucleotide Alignment Program (GSNAP) were further filtered to identify SNPs using uniquely mapped reads. SNP sites were called if they have ≥3 reads supporting it, minimum SNP base quality value ≥ 15, and rare allele coverage among all the reads must exceed 0.8 which stringently controls false SNP discovery potentially derived from sequence errors and paralogs.

#### Real-time PCR

To verify RNA-seq results, quantitative real-time PCR was conducted using SYBR green (Bio-Rad) and CFX384 Real-Time PCR detection system (Bio-Rad, Hercules, CA). SYBR® green primers for qPCR were designed using AlleleID® 7 software (Premier Biosoft, Palo Alto CA). To ensure target specificity gene sequences were blasted against non-redundant database (GenBank, NCBI) to determine cross homology with other sequences. Gene sequences were analyzed for secondary structures to avoid designing primers in these regions. Primers were designed to obtain a product between 75 and 200 bp. Primers were synthesized by Integrated DNA Technologies, Inc., San Diego, CA. Two-step RT-qPCR was performed using SYBR Green detection chemistry. cDNA was synthesized from 1 μg of total RNA and oligo(dT) primers, using the iScript™ Select cDNA Synthesis kit (Bio-Rad), following the manufacturer’s procedure. Quantitative real time PCR was carried out in a total volume of 5 uL containing 0.5 uL of template and 4.5 uL of master mix. The following amplification program was used: denaturation at 95°C for 30 s, 40 cycles of amplification (95°C for 10 s, 60°C for 30 s) and a melting curve program (from 65°C to 95°C, with an increment of 0.5°C for 5 s). Three reference genes (Additional file 2: Table S1) were used to normalize expression and these values were then compared to reads per kilobase of exon per million mapped reads (RPKM) estimates. All PCR reactions were done in triplicate on 384-well full-skirt PCR plates (USA Scientific, Ocala, FL).

## Abbreviations

DAP: Days after pollination;qRT-PCR: Quantitative real time polymerase chain reaction;ABA: Abscisic Acid;GA: Gibberllic Acid;GSNAP: Genomic short-read nucleotide alignment program;FC: Fold-changes;GO: Gene Ontology;RPKM: Reads per kilobase of exon per million mapped reads;TCA: Tricaboxylic Acid Cycle;TAG: Triacylglycerol;LTP: Lipid transfer proteins;LEA: Late embryogenesis abundant;glb2: Globulin 2 gene;SNP: Single nucleotide polymorphism

## Competing interests

The authors declare that they have no competing interests.

## Authors’ contributions

KT: Experimental design, execution and data analysis, manuscript preparation; DVR: some PCR and manuscript preparation; SD: some PCR and manuscript preparation; DJ: assistance with data analysis; XH: assistance with data analysis; JH: assistance with project development; EH: project development, design and funding. All authors read and approved the final manuscript.

## Authors’ information

EEH has worked in foreign gene expression in maize embryos for 15 years and is interested in embryo development as it informs protein accumulation.

## Supplementary Material

Additional file 1: Figure S1 The proportion of differentially expressed genes at each time point. Click here for file

Additional file 2: Table S1 qRT PCR of selected genes. Click here for file

Additional file 3: Table S2 Functional categories. Click here for file

Additional file 4: Figure S2. Gene distribution in each functional category and number of transcripts detected. Click here for file

Additional file 5: Table S3. GO terms found in embryo transcriptome. Click here for file
