# Supplementary figures and images for: Transcriptome analysis of embryo maturation in maize
Source: BMC Plant Biol. 2013 Feb 4;13:19. doi: 10.1186/1471-2229-13-19 (PMC3621147; doi:10.1186/1471-2229-13-19)

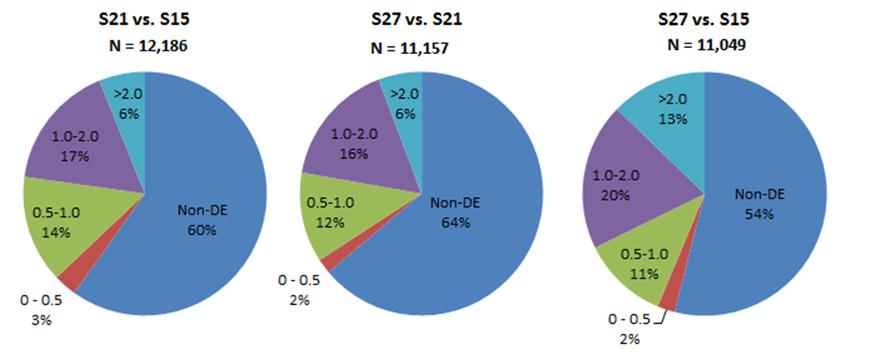

Supplement: Additional file 1: Figure S1 — The proportion of differentially expressed genes at each time point. [file 1471-2229-13-19-S1.png]

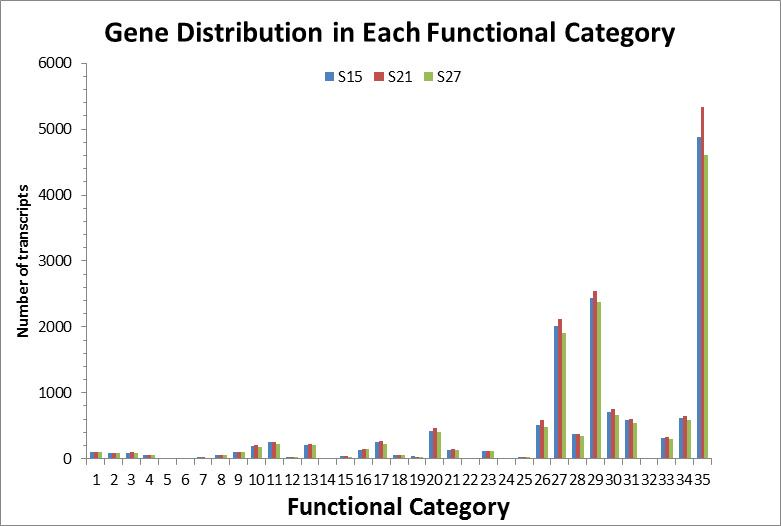

Supplement: Additional file 4: Figure S2. — Gene distribution in each functional category and number of transcripts detected. [file 1471-2229-13-19-S4.png]
