# Supplementary material for: Transcriptome analysis of embryo maturation in maize
Source: BMC Plant Biol. 2013 Feb 4;13:19. doi: 10.1186/1471-2229-13-19 (PMC3621147; doi:10.1186/1471-2229-13-19)
Supplement: Additional file 2: Table S1 — qRT PCR of selected genes. [file 1471-2229-13-19-S2.docx]

Supplemental Table 1: qRT PCR of selected genes.
